# An experimental workflow for investigating anoikis resistance in cancer metastasis

**DOI:** 10.14440/jbm.2024.0140

**Published:** 2025-08-05

**Authors:** Xue Han, Yipan Zheng, Xiaohui Si, Zhe Liu

**Affiliations:** Zhejiang Key Laboratory of Medical Epigenetics, School of Medicine, Hangzhou Normal University, Hangzhou 311121, China

**Keywords:** Anoikis resistance, Cancer metastasis, Three-dimensional spheroid culture, Circulating tumor cell assay

## Abstract

**Background:**

Anoikis is a form of programmed cell death triggered by the detachment of cells from the extracellular matrix. Anoikis resistance represents a critical factor in tumor metastasis, and elucidating the mechanisms by which epithelial cancer cells evade this process may provide a molecular insight for effectively targeting metastatic progression.

**Methods:**

Presented here are an experimental workflow and a detailed protocol to examine anoikis sensitivity in tumor cells both *in vitro* and *in vivo*. We described a detachment-induced anoikis model, a three-dimensional spheroid culture system, and an *in vivo* circulating tumor cell assay, by using the human lung carcinoma cell line A549 as a model system. We detailed the cell culture conditions, materials, and sample preparation, and the evaluation and quantification of anoikis. Together, these methods provide a comprehensive approach for investigating anoikis resistance.

**Conclusion:**

This protocol offers valuable insights into the mechanisms underlying anoikis resistance and may facilitate the identification of novel therapeutic targets for cancer treatment.

## 1. Introduction

Anoikis is a form of programmed cell death triggered by the detachment of cells from the extracellular matrix or neighboring cells.^1^,^2^ It is functionally a protective process that eliminates cells that have lost matrix attachment, thereby maintaining tissue homeostasis and preventing colonization at distant sites.^3^ Mounting evidence shows that anoikis resistance is a pivotal factor contributing to tumor metastasis.^4^-^6^

Anoikis resistance is a hallmark of malignancy, enabling cancer cells to survive in the circulatory system and colonize secondary sites – a defining feature of metastatic progression.^7^,^8^ During epithelial-mesenchymal transition and acquisition of invasive properties, cancer cells often lose their adhesive capacity and become susceptible to anoikis. Nonetheless, cancer cells can evade anoikis and metastasize through adaptive mechanisms, including alterations in integrin expression,^9^-^11^ activation of pro-survival signaling pathways,^7^,^12^ and metabolic reprogramming.^13^-^15^ Understanding the molecular mechanisms underlying anoikis resistance is crucial for the development of effective anti-metastatic therapies.^16^,^17^

In this study, we presented three experimental models and a detailed workflow for assessing anoikis sensitivity in tumor cells. This protocol involves step-by-step procedures for cell culture conditions, material and sample preparation, and the evaluation and quantification of anoikis. Collectively, this workflow offers a comprehensive framework to investigate anoikis resistance and its underlying molecular mechanisms.

## 2. Materials and methods

### 2.1. Reagents and supplies

- Dulbecco’s Modified Eagle Medium (C11995500BT, Gibco, USA)
- RPMI-1640 (C11875500BT, Gibco, USA)
- Minimum Essential Medium (10-009-CV, Corning, USA)
- Fetal bovine serum (35-015-CV, Corning, USA)
- Penicillin-streptomycin (10000 U/mL) (15140122, Gibco, USA)
- Sodium chloride (71376, Sigma-Aldrich, USA)
- Disodium phosphate (795410, Sigma-Aldrich, USA)
- Disodium phosphate (71496, Sigma-Aldrich, USA)
- Glycine (G8898, Sigma-Aldrich, USA)
- Bovine serum albumin (Fraction V) (A602440, Sangon, China)
- Glycine (G7126, Sigma-Aldrich, USA)
- Glycerol (G5516, Sigma-Aldrich, USA)
- Triton™ X-100 (T8787, Sigma-Aldrich, USA)
- Tween^®^ 20 (11332465001, Sigma-Aldrich, USA)
- 4% Paraformaldehyde (P1110, Solarbio, China)
- Cell Death Detection enzyme-linked immunosorbent assay (ELISA) PLUS kit (11774425001, Roche, Switzerland)
- Annexin V-FITC apoptosis detection kit (AT105, MULTI SCIENCES, China)
- FACS buffer (phosphate-buffered saline [PBS] with 0.5 – 1% BSA or 5 – 10% fetal bovine serum [FBS], 0.1% NaN_3_).
- TruStain FcX™ for mouse cells (156603, BioLegend, USA).
- Red cell lysis buffer (420301, BioLegend, USA).
- Corning Matrigel matrix (356234, Corning, USA)
- Phosphate-buffered saline (21-040-CV, Corning, USA)
- 0.25% Trypsin-ethylenediaminetetraacetic acid (EDTA) (25-053-Cl, Corning, USA)
- F(Ab)2 (M35200, Jackson Immunochemicals, USA)
- Secondary antibody conjugated to a fluorophore (A21422 and A11001, Thermo Fisher, USA)
- 4’,6-diamidino-2-phenylindole (DAPI) (D1306, Invitrogen, USA)
- Anti-Bcl-2 antibody (ab32124, Abcam, UK)
- Cleaved poly (ADP-ribose) polymerase 1 (PARP-1) p25 Rabbit mAb (A19612, ABclonal, China)
- PARP-1 Rabbit mAb (A19596, ABclonal, China)
- Alexa Fluor® 488 anti-human leukocyte antigen (HLA) E + HLA Class 1 ABC antibody (ab242064, Abcam, UK)
- Anti-laminin alpha 5/LAMA5 antibody (ab184330, Abcam, UK)
- Anti-integrin beta-1 antibody (ab30394, Abcam, UK)
- Nunc™ EasYFlask™ cell culture flasks (156340, Thermo Scientific, USA)
- TC-treated 24-well cell culture plates (702001, NEST, China)
- Ultra-low attachment multiple well plate (CLS3473, Corning, USA)
- EDTA tubes (HR10013, LENGRUI, China)
- 15 mL centrifuge tubes (601001, NEST, China).

### 2.2. Recipes

**Table:** 

10×PBS: Glycine (50 mL)	

Materials	Amount
NaCl	3.8 g
Na_2_HPO_4_	0.938 g
NaH_2_PO_4_	0.207 g
Glycine	3.75 g
Adjust pH to 7.4 and filter impurities	

**10×IF-wash (50 mL)**	

**Materials**	**Amount**

NaCl	3.8 g
Na_2_HPO_4_	0.938 g
NaH_2_PO_4_	0.207 g
BSA (fraction V)	5 g
Triton™ X-100	1 mL
Tween^®^ 20	0.25 mL
Adjust pH to 7.4 and filter impurities	

**Primary block**	

**Materials**	**Volume**

Phosphate-buffered saline	950 μL
Normal goat serum	50 μL
Total	1 mL

**Secondary block**	

**Materials**	**Volume**

Primary block	990 μL
F (Ab)_2_	10 μL
Total	1 mL

### 2.3. Equipment

- Centrifuge (5910R, Eppendorf, Germany)
- Carbon dioxide (CO_2_) incubator (MCO-18AIC, Panasonic, Japan)
- ELISA reader (Synergy Neo2, BioTek, USA)
- Confocal microscope (LSM710NLO, Zeiss, Germany)
- Flow cytometer (LSRFortessa, BD Biosciences, USA).

### 2.4. Software packages

- GraphPad Prism (Version 10.1.2)
- FlowJo (Version 10.8.1)
- ImageJ.

## 3. Procedures

### 3.1. Detachment-induced anoikis

#### 3.1.1. Cell preparation

**Figure 1:**
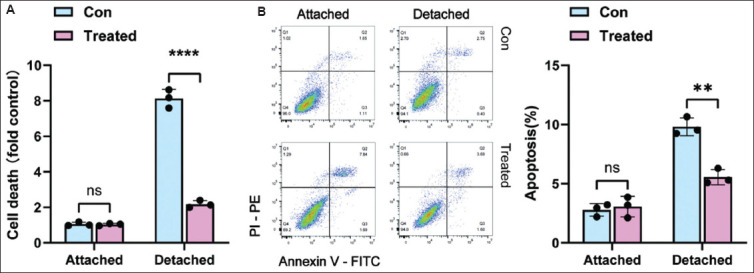
Detection of detachment-induced anoikis. (A) Cell death in control and treated A549 cells after 24 h of incubation under either attached or detached conditions. (B) Assessment of anoikis through Annexin V binding and propidium iodide uptake. Data are presented as mean ± standard deviation (SD) from three replicates in a single experiment. Note: Double asterisks (**) indicate *p*<0.01, while four asterisks (****) represent *p*<0.0001. Abbreviation: Con: Control.

1. Culture the human lung carcinoma cell line A549 in a T75 flask containing RPMI-1640 medium supplemented with 10% FBS at 37°C in a CO_2_ incubator. Monitor cell growth on a daily basis and passage the cells when they reach 70 – 80% confluence
2. Remove the culture medium and wash the cells with PBS. Add a sufficient volume of trypsin-EDTA solution to cover the cell monolayer, and incubate the cells at 37°C in a CO_2_ incubator until the cells detach (typically 1 – 3 min)
3. Neutralize the trypsin by adding an equal volume of culture medium containing FBS. Gently pipette the cell suspension to ensure single-cell dispersion. Centrifuge the cell suspension at 200 × g for 5 min. Discard the supernatant and resuspend the cell pellet in fresh medium at a concentration of 1 × 10^5^ cells/mL
4. Transfer 5 × 10^4^ cells/mL^a^ in 500 μL of medium into either ultra-low attachment or standard 24-well plates. Prepare five replicate wells for each experimental group: Two wells for cell counting and three wells for cell death detection. Incubate the cells at 37°C in a CO_2_ incubator for the desired time intervals^b^ (*e*.*g*., 0, 24, 48, and 72 h) to allow for anoikis induction.
5. Evaluate anoikis using the Cell Death Detection ELISA PLUS kit by following the manufacturer’s protocol with modifications as needed (Figure 1A).

Notes: ^a^Seeding densities in 24-well plates should be empirically determined for each cell line based on their documented doubling times and growth characteristics. Standardized seeding densities for established cell lines include: 5 × 10^4^ cells/mL for A549 and 5 × 10^4^ cells/mL for H460. ^b^For initial experiments, it is recommended to perform pilot tests using wild-type cells at various time points under both adherent and suspension conditions, since different cell lines exhibit varying degrees of resistance to anoikis.

#### 3.1.2. Solution preparation

1. Anti-Histone–Biotin: Reconstitute the vial in 450 μL of double-distilled water. Let stand for 10 min, then mix thoroughly
2. Anti-DNA–POD: Reconstitute the vial in 450 μL of double-distilled water. Let stand for 10 min, then mix thoroughly
3. Immunoreagent preparation: Combine 40 μL of Anti-Histone–Biotin solution and 40 μL of Anti-DNA–POD solution with 720 μL of incubation buffer. Mix the solution thoroughly
4. ABTS tablets: Dissolve one ABTS tablet in 5 mL of substrate buffer. Ensure complete dissolution by gentle mixing if necessary
5. ABTS Stop Solution: Allow to reach room temperature and shake gently until the solution becomes clear.

#### 3.1.3. Sample preparation

1. For attached culture plates: Digest cells from two wells using 250 μL of 0.25% trypsin-EDTA. Incubate at 37°C for 2 min. Neutralize the trypsin with an equal volume of culture medium containing FBS. Gently pipette to create a single-cell suspension. Count the cells from each well twice. Add 200 μL of lysis buffer directly to three additional wells for cell lysis.
2. For detached culture plates: Collect cells into 1.5 mL centrifuge tubes and centrifuge at 200 × g for 10 min to pellet the cells. Remove the supernatant and digest cells from the two tubes with 250 μL of 0.25% trypsin-EDTA for subsequent cell counting. Resuspend cells from three additional tubes in 200 μL of lysis buffer for cell lysis.
3. Cell lysis: Incubate the lysed cells on a shaker at room temperature at 300 rpm for 30 min. Transfer the lysate into 1.5 mL centrifuge tubes and centrifuge at 200 × g for 10 min. Collect the resulting supernatant for the assay.

#### 3.1.4. ELISA

1. Sample loading: Carefully transfer 20 μL of the collected supernatant into each well of a streptavidin-coated 96-well microplate
2. Immunoreagent addition: Slowly add 80 μL of prepared immunoreagent (4 μL of Anti-Histone–Biotin, 4 μL of Anti-DNA–POD, and 72 μL of incubation buffer) to each well
3. Incubation: Cover the plate with adhesive sealing film. Incubate on a microplate shaker at 300 rpm for 2 h at room temperature
4. Washing: Remove the solution thoroughly by tapping the plate. Wash each well 3 times with 200 μL of incubation buffer, ensuring complete removal of solution after each wash
5. Color development: Add 100 μL of ABTS solution to each well. Incubate on a shaker at 300 rpm for approximately 5 – 10 min, or until sufficient color develops
6. Reaction stoppage: Add 100 μL of ABTS stop solution to each well
7. Absorbance measurement: Measure the absorbance at 405 nm, using 490 nm as a reference wavelength. Include blank wells containing 20 μL of incubation buffer, 80 μL of ABTS solution, and 100 μL of ABTS Stop Solution. Perform measurements in duplicate to ensure accuracy.

#### 3.1.5. Analysis of assay data

Subtract the optical density value at 490 nm from the optical density value at 405 nm. Then, calculate the average values of the duplicate readings to obtain the sample absorbance value.

The following formulas can be used to quantify cell death:


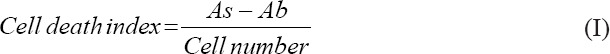



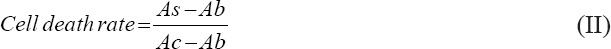


where:

(i) As = absorbance of the sample well (treated group)
(ii) Ac = absorbance of the control well (untreated group)
(iii) Ab = absorbance of the blank well (no cell lysate).

#### 3.1.6. Optional assay

*(A) Flow cytometrical analysis of apoptosis*

To evaluate apoptosis, collect anoikis-induced cells by centrifugation at 200 × g for 5 min. Resuspend the resulting cell pellet in 100 μL of binding buffer provided in the Annexin V-FITC apoptosis detection kit. Add 5 μL of Annexin V-FITC and 5 μL of propidium iodide (PI) to the cell suspension, and incubate the mixture in the dark for 15 min at room temperature. Analyze the stained cells on a flow cytometer to identify and quantify apoptotic populations. Early apoptotic cells are characterized by positivity for Annexin V and negativity for PI, while late apoptotic or necrotic cells are Annexin V-positive and PI-positive. Use flow cytometrical data to determine the percentage of cells in each population (Figure 1B).

*(B) Western blotting analysis of apoptosis*

For a molecular assessment of apoptosis, harvest cells and prepare lysates according to standard western blotting protocols. Key markers of apoptosis include cleaved caspases (*e*.*g*., caspase-3, caspase-7), Bcl-2 family proteins (*e*.*g*., Bax, Bcl-2), and cleaved PARP-1. Proteins should be separated by Sodium Dodecyl Sulfate Polyacrylamide Gel Electrophoresis, transferred onto a membrane, and probed with specific primary antibodies. Signal detection using HRP-conjugated secondary antibodies allows for the visualization and quantification of apoptotic markers.

### 3.2. 3D spheroid culture

#### 3.2.1. Material preparation

1. Put aliquot Matrigel Matrix into 1.5 mL centrifuge tubes and store at −20°C for later use. Thaw one vial of Matrigel Matrix overnight at 4°C, swirling gently to ensure uniform dispersion. Keep the Matrigel Matrix on ice until ready for use. Ensure all materials that come into contact with Matrigel, including culture plates and pipette tips, are pre-chilled at −20°C before use
2. Prepare RPMI-1640 or DMEM medium containing 5% Matrigel and 2% FBS, and store at 4°C. For 10 mL of medium, mix 500 μL of Matrigel, 200 μL of FBS, and 9.3 mL of RPMI-1640 or DMEM. Keep all reagents on ice before use.

#### 3.2.2. Cell preparation

1. Wash A549 cells with PBS, digest them with trypsin, and neutralize the trypsin by adding an equal volume of culture medium containing FBS. Centrifuge the suspension at 200 × g for 5 min
2. Resuspend the cells in complete medium (containing 2% FBS^c^) to a final density of 2 × 10^5^ cells/mL. Mix 5 μL of the cell suspension with 500 μL of RPMI-1640 medium containing 5% Matrigel and 2% FBS. Pipette the mixture thoroughly and plate 1 × 10^4^ cells/mL^d^ per well in either low-adhesion 24-well plates or pre-coated 24-well plates
3. Incubate the cells at 37°C in a CO_2_ incubator for 7 – 14 days. Replace the medium with fresh RPMI-1640 containing 5% Matrigel and 2% FBS every 3 days
4. Observe the cultures under an inverted microscope, focusing on five random fields per well at 100× to 200× magnifications. Count colonies with a diameter greater than 50 μm. Calculate the average colony number per field across triplicate wells and express the results as mean ± standard error of the mean. Capture the images as needed.

Notes: ^c^For more fastidious cell types or primary cultures, increasing the FBS concentration may be beneficial. ^d^Cell numbers should be optimized based on the proliferation rate of each cell line, ideally ensuring that spheroids originate from single, independent clones.

#### 3.2.3. Immunofluorescence staining

**Figure 2:**
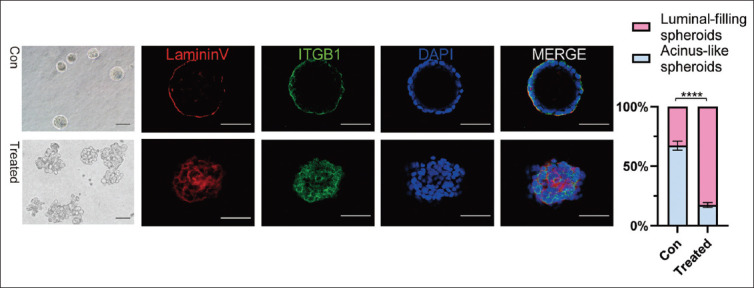
Three-dimensional spheroid culture of A549 cells. Control and treated A549 cells were embedded in Matrigel and cultured for 8 days. Representative confocal midplane sections are shown, immunostained for laminin V (red), ITGB1 (green), and DAPI (blue). Colonies larger than 50 μm in diameter were counted. Scale bars represent 50 μm. Data are presented as mean ± standard deviation for 10 visualized areas in a single experiment. Note: Four asterisks (****) denote *p*<0.0001. Abbreviations: Con: Control; ITGB1: Integrin beta-1.

1. Wash and dry the slides. Outline the region for spheroid placement using a PAP pen
2. Collect spheroids into 15 mL centrifuge tubes and centrifuge at 1,000 rpm for 1 min^e^
3. Discard the supernatant, leaving approximately 100 μL of liquid. Gently mix and dispense the spheroids onto a prepared slide
4. Allow the sample to air-dry for approximately 10 min, then fix with 2% paraformaldehyde at room temperature for 30 min. Wash the slides 3 times with PBS to remove residual fixative, air-dry, and store at −20°C for later use
5. Rinse the slides with 1 × PBS-Glycine 3 times for 10 min each
6. Permeabilize cells by applying permeabilization buffer and incubate at room temperature for 5 – 10 min
7. Wash 3 times with 1× IF wash buffer, for 5 min per wash
8. Block non-specific binding by incubation with blocking buffer for 1 h at room temperature. Agitate gently to ensure uniform blocking. Perform an additional blocking step for 40 min at room temperature
9. Dilute the primary antibody (*e*.*g*., anti-laminin V or ITGB1) in blocking buffer to the recommended concentration
10. Apply the diluted primary antibody and incubate overnight at 4 °C or for 2 h at room temperature. Wash the slides 3 times with 1× IF wash buffer for 20 min each
11. Add the diluted secondary antibody and incubate for 1 h at room temperature in the dark. Wash 3 times with 1× IF wash buffer for 20 min each
12. Stain nuclei with a nuclear stain (*e*.*g*., DAPI) for 5 – 10 min at room temperature. Wash 3 times with PBS to remove excess stain
13. Mount the slides using 90% glycerol in PBS
14. Visualize and capture images of the 3D cultures using a confocal microscope (Figure 2).

Notes: Perform all steps with minimal light exposure to prevent photobleaching of fluorophores. ^e^Cell spheroids are typically visible. Centrifugation speed may be moderately increased to ensure complete pelleting while maintaining spheroid integrity.

### 3.3. *In vivo* circulating tumor cell assay

#### 3.3.1. Cell culture and xenograft model

1. Culture A549 cells in RPMI-1640 medium supplemented with 10% FBS at 37°C in a humidified incubator containing 5% CO_2_
2. Mix 5 × 10^5^ cells/mL of A549 with 2.5 × 10^5^ cells/mL of human lung cancer-associated fibroblasts in 120 μL of PBS containing Matrigel (1:1, v/v)
3. Inject the cell suspension subcutaneously into the flanks of 8-week-old female BALB/c nude mice, with five mice per group
4. Collect 100 μL of peripheral blood from each mouse eight weeks post-inoculation for circulating tumor cell analysis.

#### 3.3.2. Red blood cell lysis

1. Isolate peripheral blood mononuclear cells (PBMCs) containing circulating tumor cells using red blood cell lysis buffer. To lyse red blood cells, add 1 mL of lysis buffer to 100 μL of peripheral blood, mix gently, and incubate for 5 min at room temperature
2. Dilute the lysis mixture with 10 mL of medium or PBS containing 2% FBS. Then, centrifuge at 400 × g for 10 min at 4°C
3. Discard the supernatant and retain the cell pellet for further analysis.

#### 3.3.3. Staining and flow cytometrical analysis

**Figure 3:**
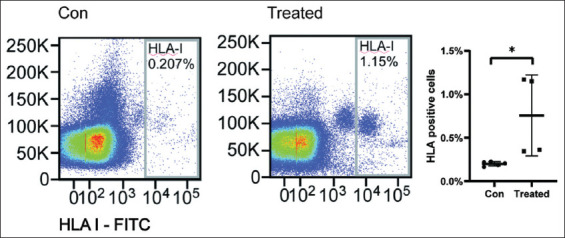
*In vivo* circulating tumor cell assay. Peripheral blood samples were harvested from mice and processed for HLA-I immunostaining. The frequency of HLA-I-positive circulating tumor cells was flow cytometrically quantified. Abbreviations: Con: Control; HLA: Human leukocyte antigen.

1. Resuspend the pelleted cells in 100 μL of ice-cold FACS buffer. To minimize non-specific binding, add 5 μL of TruStain FcX™ per million cells in a 100 μL staining volume. Incubate for 10 min at room temperature
2. Add FITC-conjugated anti-HLA-I antibodies to each sample to distinguish human tumor cells from mouse blood cells, and incubate for 20 min in the dark at room temperature
3. After staining, centrifuge the cells at 400 × g for 10 min at 4°C and wash once with at least 2 mL of ice-cold FACS buffer. Then, resuspend the washed cells in 300 μL of ice-cold FACS buffer for flow cytometrical analysis (Figure 3). Use PBMCs from tumor-free mice as negative controls to define the gating parameters for FITC-positive tumor cells.

Keep all reagents and solutions ice-cold, and perform staining at 4°C to prevent surface antigen modulation and internalization. If immediate analysis is not feasible, store the samples in the dark at 4 – 8°C in a fixation buffer containing 2% paraformaldehyde in PBS.

## 4. Discussion

The experimental workflow presented here detailed methodologies to characterize anoikis resistance in cancer cells. In the detachment-induced anoikis model, the use of the Cell Death Detection ELISA PLUS kit and flow cytometry allowed for precise quantification of cell death, revealing that treated A549 cells exhibited anoikis resistance. The 3D spheroid culture system further confirmed that treated A549 cells formed solid, spheroidal colonies, indicating their ability to survive in an anchorage-independent manner – a hallmark of metastatic potential. Furthermore, the *in vivo* circulating tumor cell assay identified HLA-I-positive human tumor cells in the mouse peripheral blood, confirming that A549 cells can evade anoikis in the circulatory system and potentially initiate distant metastases. Notably, the findings from cell apoptosis assays following detachment indicate that tumor cells undergoing anoikis exhibit diverse modes of cell death, with apoptosis accounting for a relatively low proportion. The consistency between *in vitro* and *in vivo* results underscores the translational value of these methods.

## 5. Conclusion

This protocol provides a comprehensive experimental framework for investigating anoikis resistance by integrating *in vitro* and *in vivo* models to elucidate key mechanisms underlying cancer metastasis. By enabling systematic analysis of cell survival pathways, these methods may facilitate the discovery of novel therapeutic targets for metastatic diseases.

## Data Availability

Not applicable.
